# Poly-4-Hydroxybutyrate as a Novel Biomaterial in Personalized Breast Surgery: A Systematic Review and Meta-Analysis

**DOI:** 10.3390/jpm15080368

**Published:** 2025-08-12

**Authors:** Joseph M. Escandón, Ajani Nugent, Nolan S. Karp, Krishna Vyas, Carter J. Boyd, Lucas Kreutz-Rodrigues, Oscar J. Manrique

**Affiliations:** 1 Department of Surgery, Wyckoff Heights Medical Center, 374 Stockholm St., New York, NY 11237, USA; anugent@wyckoffhospital.org; 2 Hansjörg Wyss Department of Plastic Surgery, New York University Langone Health, New York, NY 10016, USA; nolan.karp@nyulangone.org (N.S.K.);; 3 Private Practice, 800 A 5th Ave., Suite 300 A, New York, NY 10065, USA; vyasplastics@gmail.com; 4 Division of Plastic Surgery, Mayo Clinic, 200 1st St. SW, Rochester, MN 55905, USA; kreutzrodrigues.lucas@mayo.edu; 5 Austin-Weston Center for Cosmetic Surgery, 1825 Samuel Morse Dr., Reston, VA 20190, USA; oscarj.manrique@gmail.com

**Keywords:** “tissue scaffolds” [Mesh], “breast” [Mesh], “mammaplasty” [Mesh], “breast implantation” [Mesh]

## Abstract

**Background/Objectives**: In the search for optimal meshes and matrices in breast surgery, poly-4-hydroxybutyrate (P4HB) has emerged as a promising alternative. This review evaluates the clinical application of P4HB scaffolds, focusing on complication rates and surgical outcomes. **Methods**: A systematic search was conducted using PubMed and ScienceDirect. Clinical studies assessing perioperative outcomes and complications associated with P4HB scaffolds in breast surgery were included. Results were stratified into aesthetic and reconstructive surgery categories. Meta-analysis was implemented to assess the rate of complications and satisfaction. **Results**: This systematic review included 13 studies evaluating the use of P4HB scaffold in breast reconstruction (636 cases) and aesthetic breast surgery (462 patients). Breast reconstruction studies were all retrospective, mainly reporting two-stage, prepectoral, immediate reconstructions. Aesthetic studies included both prospective and retrospective designs, with varied implant planes and incision patterns. P4HB use was associated with high satisfaction (95.5%) and favorable outcomes, including lower odds of wound complications (log-OR = −1.135, *p* = 0.003). Complication rates were low across both surgical categories. P4HB scaffold showed promise in supporting implant-based procedures and maintaining breast shape over time, with minimal increase in surgical time and stable anthropometric measurements. **Conclusions**: The use of P4HB scaffold in breast reconstruction and aesthetic surgery shows promising results, notably in reducing wound-related complications. Breast reconstruction studies report low complication rates and favorable patient-reported outcomes. In aesthetic procedures, P4HB contributes to improved long-term breast shape and high satisfaction. Despite encouraging findings, further research is necessary to validate long-term efficacy and refine surgical approaches.

## 1. Introduction

The field of breast surgery has undergone substantial evolution with the integration of biomaterials designed to provide soft tissue reinforcement and structural support. These innovations include synthetic meshes, acellular dermal matrices (ADMs), dermal substitutes, and biosynthetic scasffolds. Among these, poly-4-hydroxybutyrate (P4HB)—a resorbable monofilament scaffold—has emerged as a promising material for both reconstructive and aesthetic breast surgery [1,2].

ADMs and synthetic meshes were initially adopted to support implant-based breast reconstruction, facilitating both submuscular and prepectoral placement [3,4,5,6]. These materials enabled the creation of a stable prosthetic pocket by securing the mesh or ADM between the inferior border of the pectoralis major muscle and the inframammary fold (IMF) [3,4]. This approach often eliminated the need for tissue expansion, allowing for direct-to-implant reconstruction. Over time, their use has been associated with improved outcomes, including reduced capsular contracture rates and enhanced pocket stability [7,8,9].

Despite these advantages, complications associated with biological and synthetic scaffolds, such as postoperative pain, edema [10], mastitis [10], chronic abscess formation [11], seroma, and implant failure—have prompted continued investigation. At times, this has led to increased risk of infection or mesh removal [12,13,14,15,16]. In response, P4HB has garnered attention as a next-generation scaffold with potential to mitigate many of these issues.

P4HB belongs to the polyhydroxyalkanoate family, a class of naturally occurring biopolymers known for their biocompatibility and favorable mechanical properties [2,17]. Engineered to retain tensile strength throughout the critical wound-healing phase, P4HB offers mechanical integrity three to five times greater than native tissue [18]. Given these characteristics, P4HB-based scaffolds represent a novel and personalized approach to addressing the challenges associated with soft tissue reinforcement in breast surgery [18]. This systematic review and meta-analysis comprehensively assess the clinical application of P4HB scaffolds in both aesthetic and reconstructive breast surgery. Specifically, we evaluate the incidence of complications, characterize surgical outcomes, and examine patient-reported satisfaction following the use of P4HB in breast surgery.

## 2. Materials and Methods

### 2.1. Literature Search

This article adheres to the Declaration of Helsinki and conforms to the Preferred Reporting Items for Systematic Reviews and Meta-Analyses (PRISMA) guidelines [19,20]. A comprehensive search of PubMed (MEDLINE), Cochrane Library (John Wiley & Sons, Inc., Hoboken, NJ, USA), and ScienceDirect (Amsterdam, The Netherlands) [Elsevier, B.V.] databases was conducted from inception through 10 June 2025. The search strategy included combinations of Medical Subject Headings (MeSH) and free-text terms as follows: ((“Hydroxybutyrates” [Mesh]) OR (“poly(4-hydroxybutanoate)” [Supplementary Concept]) OR (4-hydroxybutyrate) OR (Poly-4-Hydroxybutyrate) OR (GalaFLEX) OR (P4HB) OR (Phasix)) AND ((“Surgical Mesh” [Mesh]) OR (“Breast Implants” [Mesh]) OR (“Mammaplasty” [Mesh]) OR (“Breast Implantation” [Mesh]) OR (Breast)). Reference lists of eligible articles were manually reviewed to identify additional studies.

### 2.2. Types of Studies

Eligible studies included retrospective and prospective case series, case-control studies, cohort studies, and single- or double-arm randomized clinical trials that reported perioperative outcomes and complications associated with P4HB scaffolds in breast surgery. Exclusion criteria included animal studies, review articles, case reports, conference abstracts, posters, editorials, letters, protocols, guidelines, and prior systematic reviews. No restrictions were placed on language or date of publication. Two reviewers (J.M.E., O.J.M.) independently screened all titles and abstracts, followed by full-text review of potentially eligible articles. Any discrepancies were resolved through consensus, involving a senior third reviewer who conducted a full-text evaluation of the articles in question. The interrater reliability was analyzed with the Cohen’s kappa (*κ*) coefficient.

### 2.3. Variables of Interest

Data were extracted independently by two reviewers using a standardized collection form. Extracted variables included the number of patients, number of reconstructions, follow-up length, age, body mass index (BMI), comorbidities (e.g., diabetes, hypertension), smoking status (current/non-smoker), adjuvant systemic chemotherapy or radiotherapy, timing of reconstruction (immediate/delayed), type of prosthesis used, laterality of reconstruction (bilateral/unilateral), type of mammaplasty procedure (reduction, mastopexy, or augmentation), indication for procedure, weight of resected specimen, type of reconstruction (two stage/direct-to-implant), type of mesh or scaffold used, plane of implant placement, and surgical technique.

Perioperative surgical outcomes analyzed in this study included surgical time, length of stay, and satisfaction. Satisfaction among patients was determined when a patient was explicitly reported to be “satisfied” or “very satisfied,” when no further reinterventions were required regarding cosmetic apprehensions, or when satisfaction was stated above a numeric threshold in a satisfaction score.

The complications analyzed for this review were as follows: return to the operating room (RTOR), infection, seroma, wound-related complications, implant exposure, explanation, palpable mesh, capsular contracture, and implant malposition. Wound-related complications comprised delayed wound healing, mastectomy flap necrosis, and dehiscence or wound break down. Only patients who required reintervention due to complications were included in the RTOR rate. Elective secondary procedures or revisions were not considered RTOR. Missing data were reported qualitatively as not available or “N/A”. Studies lacking data for a specific outcome were excluded from the corresponding quantitative analyses.

### 2.4. Statistical Analysis

Weighted means were calculated for continuous variables: (∑^n^_i=1_[xi × wi]/∑^n^_i=1_wi). For reconstructive studies, each breast was considered an individual unit of analysis. For aesthetic procedures, each patient was treated as a single unit. For single-cohort studies or case series, binominal data were analyzed, and complication rates were pooled using proportion meta-analysis.

For studies comparing procedures performed with P4HB scaffold (intervention group) to a control group (other mesh/scaffold), the analysis utilized the log odds ratio (log-OR) as the primary outcome measure. Statistical analyses were performed using Jamovi software version 1.2.27.0 (Jamovi, Sydney, Australia). A random-effects model was applied, and the level of heterogeneity—represented by tau-squared (τ^2^)—was calculated using the Sidik–Jonkman method [21]. Additional heterogeneity metrics included the Cochran’s *Q*-test and *I*^2^ statistic [22]. Heterogeneity was classified as moderate (*I*^2^ = 30–60%), substantial (50–90%), or considerable (>90%) [23]. Funnel plot asymmetry was evaluated via regression testing using standard error as a predictor [24]. We used meta-regression to explore the potential impact of type of aesthetic procedure (revision versus no revision), age, and BMI on the rate of satisfaction.

### 2.5. Risk of Bias and Quality Assessment

Risk of bias in non-randomized cohort studies was evaluated using the Risk Of Bias In Non-randomized Studies-of Interventions (ROBINS-I) tool [25,26]. Case series were assessed using the Methodological Quality Assessment Tool (MQAT) described by Murad et al. [25,26]. The level of evidence was evaluated using the Oxford Centre for Evidence-Based Medicine (OCEBM) guidelines [27].

## 3. Results

### 3.1. Included Studies

**Figure 1:**
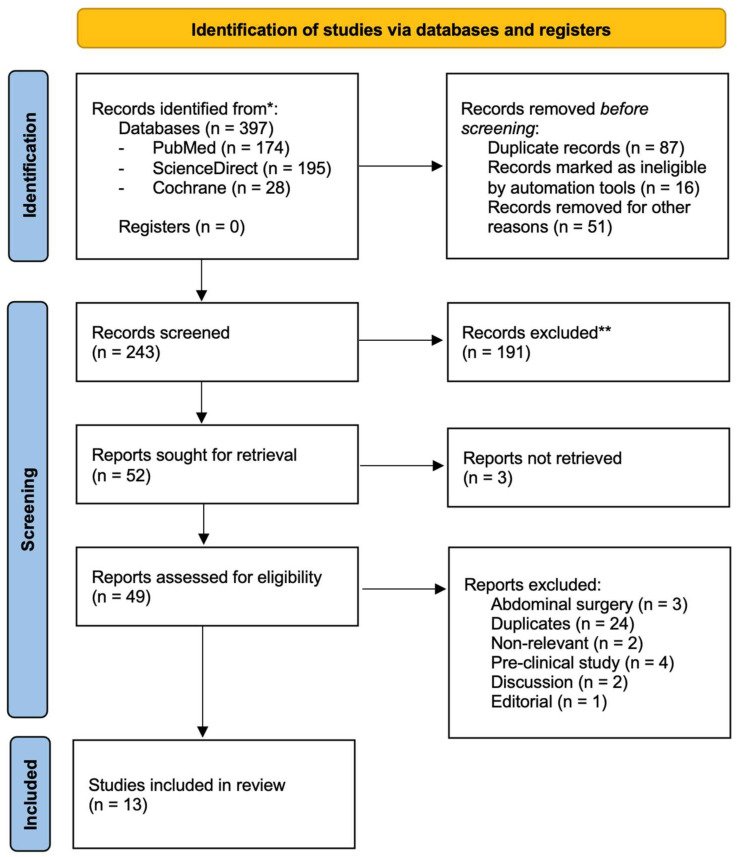
Diagram of the study selection process for the systematic review and meta-analysis. * Records identified from each database or register searched. ** Automation tools were used.

Three hundred ninety-seven references were evaluated. Two hundred forty-three citations were assessed based on their titles and abstracts after excluding duplicates. Ultimately, thirteen studies were included after full-text review (Figure 1) [1,8,12,16,17,18,28,29,30,31,32,33,34]. Interrater reliability was high, with a Cohen’s kappa of 0.859, indicating near-perfect agreement.

Five studies (38.5%) focused on breast reconstruction and reported outcomes from 636 reconstructions using P4HB scaffolds (Table 1) [12,16,17,28,29]. All studies evaluating outcomes of breast reconstruction were retrospective in design. The mean age of participants ranged from 44.8 to 53.7 years, and mean BMI ranged from 23.9 to 29.9 kg/m^2^ [12,16,17,28,29]. Two reports were single-cohort studies [17,28], while three reports were comparative studies (≥2 cohorts) [12,16,29]. Comparative studies included 929 reconstructions performed without P4HB scaffolds, using either other meshes or no mesh at all [12,16,29].

Eight studies (61.5%) evaluated P4HB use in aesthetic breast surgery, encompassing 462 patients (Table 2) [1,8,18,30,31,32,33,34]. Two studies were prospective [1,32], and six were retrospective [1,8,18,30,31,32,33,34]. The mean age ranged between 22.3 and 46.2 years, and mean BMI between 22.4 and 24.7 kg/m^2^ [1,8,18,30,31,32,33]. Two double-cohort studies were identified [1,34], while six articles were single-cohort studies [8,18,30,31,32,33]. Comparative cohorts included 40 patients as control groups who underwent aesthetic procedures without P4HB scaffolds [1,34].

**Figure 2:**
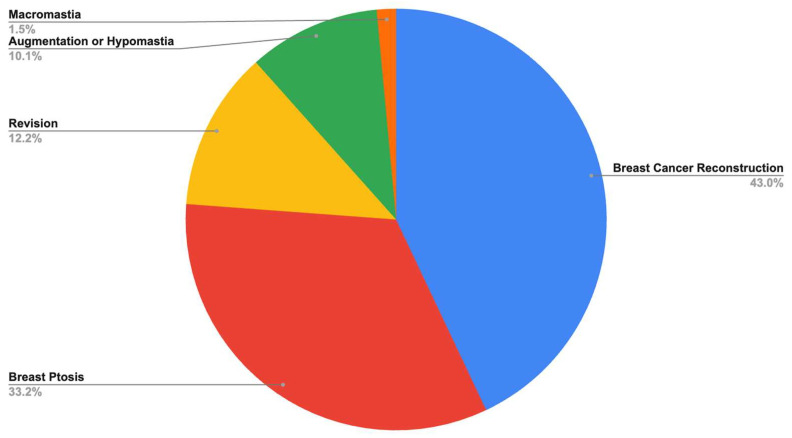
Indication for P4HB scaffold placement.

**Figure 3:**
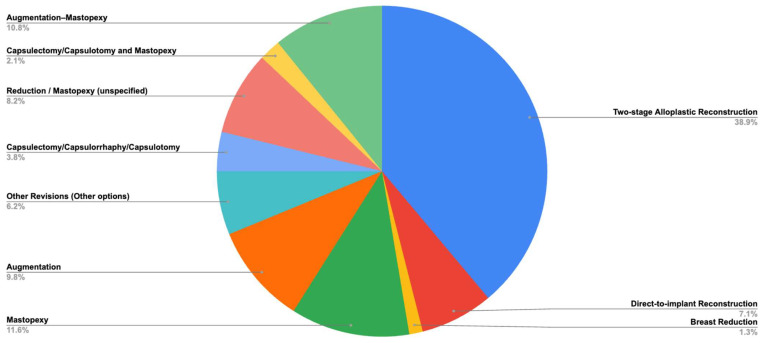
Main procedures performed at the time of P4HB scaffold placement. Statistical software automatically rounds decimal values.

Among all studies included, the most common indications for P4HB scaffold use were breast cancer reconstruction (42.96%), breast ptosis (33.21%), and revision of previous aesthetic surgeries (12.22%) (Figure 2). The most frequently reported procedures in the literature using P4HB scaffold for breast surgery were two-stage alloplastic reconstruction (38.89%), mastopexy (11.64%), augmentation–mastopexy (10.85%), and augmentation alone (9.79%) (Figure 3).

### 3.2. Breast Reconstruction Outcomes

When P4HB scaffold was used for breast reconstruction, three studies reported the laterality of breast reconstruction (Table 3) [12,17,29]. The percentage of bilateral breast reconstructions using P4HB ranged between 75% and 85.2% [12,17,29]. The average oncologic specimen weight was only reported in one study, and was 472 ± 275 g [17]. Only one study reported using intercostal nerve blocks [17]. Timing of reconstruction was reported in four studies, all of which involved immediate reconstruction [12,17,28,29]. Three studies implemented two-stage alloplastic reconstruction [12,16,17], while two reports used a direct-to-implant approach [28,29]. One study used a dual-plane technique for tissue expander placement [16], while three studies uniformly elected a prepectoral approach [12,17,28]. Diffley et al. reported 95.2% of reconstructions as prepectoral and the remainder subpectoral [29].

Chen et al. found that time to full expansion was similar between ADM and P4HB in dual-plane reconstructions: 178 ± 99.5 mL/month vs. 197 ± 135 mL/month, respectively [16]. In two-stage reconstruction, Movassaghi et al. reported a mean final implant size of 573 ± 153.4 cc, with 94.4% of reconstructions undergoing adjunctive fat grafting [17]. The surgical outcomes of reconstructions performed with and without P4HB are summarized in Table 3 [12,16,29]. Only one study evaluated patient-reported outcomes using the BREAST-Q tool, with scores of 72.3 (satisfaction), 77.2 (psychosocial well-being), 67.2 (sexual well-being), and 6.3 (satisfaction with implants) [17].

### 3.3. Breast Reconstruction Complications

Overall, the pooled incidence of periprosthetic infection was 3.8% (95% CI 1.2–6.3%) with substantial heterogeneity (*I*^2^ = 62.9%, *Q* = 9.8, *p* = 0.044). Seroma occurred in 1.3% of cases (95% CI < 0.01–2.7%) with moderate heterogeneity (*I*^2^ = 45.18%, *Q* = 6.93, *p* = 0.074). The pooled incidence of wound-related complications was 2.5% (95% CI 1.1–4%) and heterogeneity was not relevant (*I*^2^ = 21.9%, *Q* = 3.05, *p* = 0.54) (Supplemental Digital Content S1).

Given the substantial heterogeneity among studies reporting capsular contracture rates (*I*^2^ = 99.78%, *Q* = 84.60, *p* < 0.001), sensitivity analysis was conducted to evaluate the robustness of the pooled incidence estimate. One study was identified as a statistical outlier and was excluded from the analysis using the interquartile method (points above Q3 + (1.5 × IQR) are considered outliers) [16]. Following this exclusion, the pooled incidence of capsular contracture was 0.9% (95% CI: <0.01% to 1.8%), with heterogeneity markedly reduced and no longer statistically significant (*I*^2^ = 5.1%, *Q* = 0.25, *p* = 0.88) [12,17,29].

Subgroup analysis limited to studies reporting outcomes for prepectoral reconstruction revealed a pooled incidence of periprosthetic infection of 3.1% (95% CI < 0.01–6.2%) with substantial heterogeneity (*I*^2^ = 77.97%, *Q* = 6.571, *p* = 0.037). Seroma occurred in 1.3% of cases (95% CI < 0.01–3.1%) with moderate heterogeneity (*I*^2^ = 64.3%, *Q* = 6.661, *p* = 0.036). The pooled incidence of wound-related complications was 2.4% (95% CI 0.6–4.1%) and heterogeneity was moderate (*I*^2^ = 38.99%, *Q* = 2.279, *p* = 0.32) [12,17,28].

Subgroup analysis limited to studies reporting outcomes for immediate alloplastic reconstruction revealed a pooled incidence of periprosthetic infection of 3.1% (95% CI 0.6% to 5.7%) with substantial heterogeneity (*I*^2^ = 61.77%, *Q* = 6.803, *p* = 0.78). Seroma occurred in 1.3% of cases (95% CI < 0.01–2.7%) with moderate heterogeneity (*I*^2^ = 45.18%, *Q* = 6.93, *p* = 0.074). The pooled incidence of wound-related complications was 2.3% (95% CI 0.8–3.8%) and heterogeneity was not relevant (*I*^2^ = 20.82%, *Q* = 2.279, *p* = 0.517) [12,17,28,29]. The pooled incidence of capsular contracture was 0.9% (95% CI: <0.01–1.8%) and heterogeneity was not relevant (*I*^2^ = 5.1%, *Q* = 0.25, *p* = 0.88) [12,17,28].

**Figure 4:**
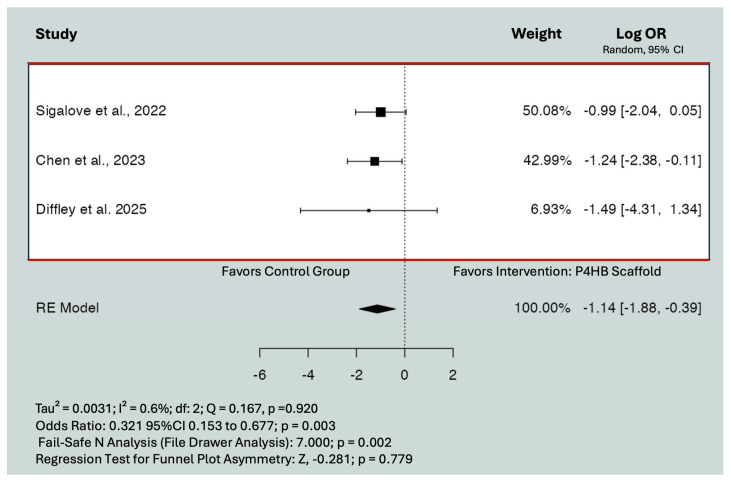
Effect of P4HB on the rate of wound-related complications. Point estimates and 95% CIs are shown (random-effects calculations for the meta-analysis). The log-ORs were −1.135 (95% CI −1.88 to −0.391; *p* = 0.003). Back transformation into odds ratios demonstrated OR: 0.321 (95% CI 0.153 to 0.677; *p* = 0.003). Dashed line: Line of no effect that represents the “null’ value, where there is no effect. Black square: Individual study result representing the point estimate. Black diamond: Pooled effect size from all studies combined [12,16,29].

The log odds ratio of recipient site infection (log-OR = −0.436; 95% CI = −1.290 to 0.419; *p* = 0.317) and capsular contracture (log-OR = 0.432; 95% CI = −0.255 to 1.119; *p* = 0.218) did not significantly decrease with the incorporation of P4HB scaffold compared to reconstructions performed with other types of mesh. Heterogeneity was not clinically relevant for any of these models. The log odds ratio of wound-related complications significantly decreased with the incorporation of P4HB scaffold (log-OR = −1.135; 95% CI = −1.88 to −0.391; *p* = 0.003) (Figure 4). Heterogeneity was not clinically relevant (*I*^2^ = 0.6%, *Q* = 0.167 *p* = 0.920). This indicated that the ratio of the probability of presenting with wound-related complications was lower with P4HB compared to other types of mesh.

### 3.4. Aesthetic Surgery Outcomes

The outcomes of P4HB scaffold in aesthetic breast surgery were reported in 462 patients (Table 4). The incision patterns were reported in four articles [1,32,34]: two studies used Wise-pattern incisions [1,34]; one used the keyhole pattern [32]; and another used inframammary (80%) or periareolar (20%) incisions [18]. Two studies recorded the weight or volume of the resected specimen [30,32]. Bistoni et al. reported a mean glandular resection weight of 286.4 g (70–653 g) [32]. The other study reported a mean glandular resection volume of 122.1 ± 110.4 cm^3^ on the right and 131.5 ± 107.8 cm^3^ on the left side [30].

Four case series reported the mean size of implants used [8,18,31,32]. The highest mean implant size was reported by Nair & Mills (540 ± 157.043 cc) [18]. Otherwise, the average implant sizes were 345 cc (325–440 cc), 244 cc (170–320 cc), and 294 cc (175–510 cc) in the other three studies [8,31,32]. Five series provided data regarding the plane for implant placement [8,18,31,32,33]. Three studies used a sub-glandular approach [8,18,31]. Nair & Mills reported that a subpectoral approach was used in 80% of the cases and sub-glandular in 20% [18], while the study by Bistoni et al. specifically indicated subfascial implant placement [32]. Sinclair & Adams reported unspecified implant exchange with site change in 12.1% of the cases and implant exchange with neo-subpectoral pocket in 6.9% of the cases [33].

**Figure 5:**
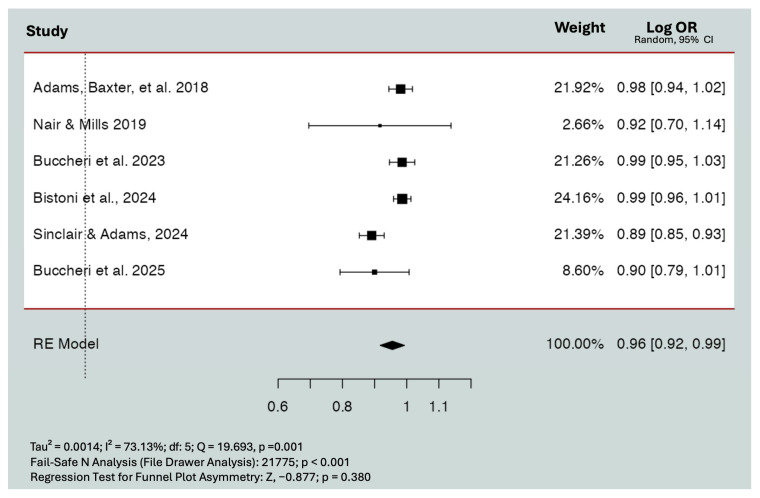
Forest plot for the meta-analysis of pooled rate of satisfaction following breast aesthetic surgery with P4HB. Black square: Individual study result representing the point estimate. Black diamond: Pooled effect size from all studies combined [1,8,18,30,32,33]. Dashed line: Line of no effect that represents the “null’ value, where there is no effect.

Two studies reported surgical time. Mean operative time for augmentation revision (capsulectomy with/without mastopexy) was 160 min (range: 140–180 min) [8]. Buccheri et al. indicated that there was no difference between mastopexy with and without P4HB for the surgical time (121.5 ± 9.2 min versus 122.5 ± 10.2 min, *p* > 0.05) [1]. The pooled satisfaction rate of aesthetic breast procedures with P4HB was 95.5% (95% CI 91.7–99.3%, *p* < 0.001) (Figure 5). Heterogeneity was substantial (*I*^2^ = 73.13%, *Q* = 19.693, *p* = 0.001). Meta-regression revealed no impact of age, BMI, or type of aesthetic procedure on the pooled satisfaction rate of aesthetic breast surgery with P4HB. The pooled satisfaction rate when P4HB was used only for primary breast augmentation, secondary breast augmentation, or breast augmentation revision was 98.5% (95% CI 95.6–99.9%, *p* < 0.001). Heterogeneity was low (*I*^2^ = 23.59%, *Q* = 0.374, *p* = 0.829) [8,18,32].

Four studies reported anthropometric measures evaluation [1,30,32,34]. Bistoni et al. found that the curved distance from the nipple to the inframammary fold (N-IMF) increased significantly over time after surgery. At 6 weeks after surgery, this distance was 7.9 cm, but it increased by 5.39% (8.4 cm) at 6 months and 8.04% (8.6 cm) at 12 months (*p* < 0.0001). However, the distance from the sternal notch to the nipple (SN-N) remained stable throughout the follow-up period (SN-N distance at 6 weeks: 21.06 cm, SN-N distance at 12 months: 21.57 cm; *p* = 0.242) [32].

When using P4HB for mastopexy using an inferior pedicle (Dermo-adipose) technique, Bucccheri et al. demonstrated a reduced mean N-IMF distance (8.47 ± 1.33 cm) compared to mastopexy without P4HB scaffold (9.57 ± 1.510.6 cm; *p* = 0.0038) at the longest follow up [1]. For breast reduction, Cagli et al., 2024 [34] reported that with P4HB scaffold, the 12-month mean N-IMF distance elongation variation was only 5.6% compared to 12% when P4HB scaffold was not employed. Adams, Baxter, et al. observed a significant redistribution of breast volume between months 3 and 12 after surgery with P4HB scaffold, with an average 11% decrease in upper pole volume and an 11.5% increase in lower pole volume, indicating a downward shift in tissue distribution [30].

### 3.5. Aesthetic Surgery Complications

For procedures involving mastopexy or breast reduction, the reported rates of complications were as follows: infection ranged from 0 to 6.4%, seroma 0%, skin flap necrosis 0%, dehiscence ranged from 0 to 11.3%, hematoma from 0 to 4.8%, and return to the operating room (RTOR) from 0 to 6.4% [1,30,34]. In cases of augmentation mastopexy, the incidence of seroma and infection was 0%, while dehiscence occurred in 4.2% and delayed wound healing in 11.1% of cases [32].

For revision procedures involving capsular work (capsulectomy or capsulotomy) with or without concurrent mastopexy, the rates of infection, seroma, wound dehiscence, hematoma, and RTOR were uniformly 0% [8,18,31] (Supplemental Digital Content S2).

### 3.6. Quality Assessment

**Figure 6:**
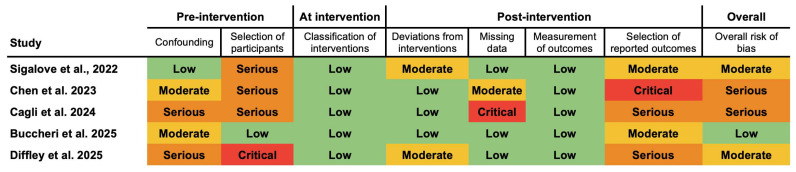
Assessment of risk of bias with ROBINS-I, a tool for assessing risk of bias in non-randomized studies of interventions. Cohen’s kappa (κ) was >0.81 for all parameters [1,12,16,29,34].

**Figure 7:**
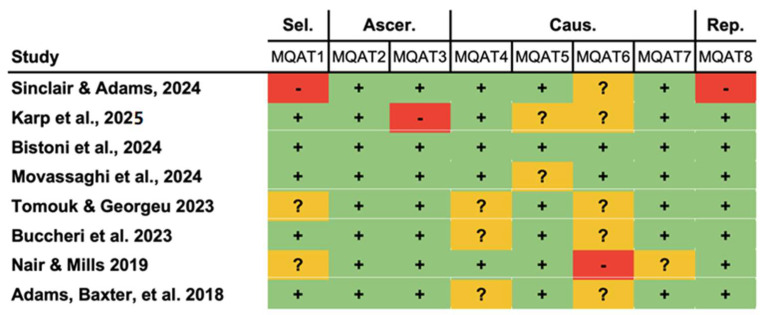
Risk of bias in case series using the Methodological Quality Assessment Tool (MQAT) [8,17,18,28,30,31,32,33].

All studies had an OCEBM level of IV (92.3%), except for one prospective study [1]. None of the studies were randomized trials. Protocols for random sequence generation and allocation concealment were not evident. None of the studies reported blinding of patients or surgeons. The assessment for ROBINS-I is exhibited in Figure 6. Employing the MQAT, one study scored 8, one scored 7, two scored 6, and four scored 5 (Figure 7). Four studies did not provide explicit data on follow-up length [16,28,30,34]. Chen et al. did not specify whether alloplastic breast reconstruction was performed in the immediate or delayed setting [16]. Similarly, Diffley et al. did not distinguish outcomes based on the reconstructive plane, i.e., prepectoral versus subpectoral placement [29].

**Figure 8:**
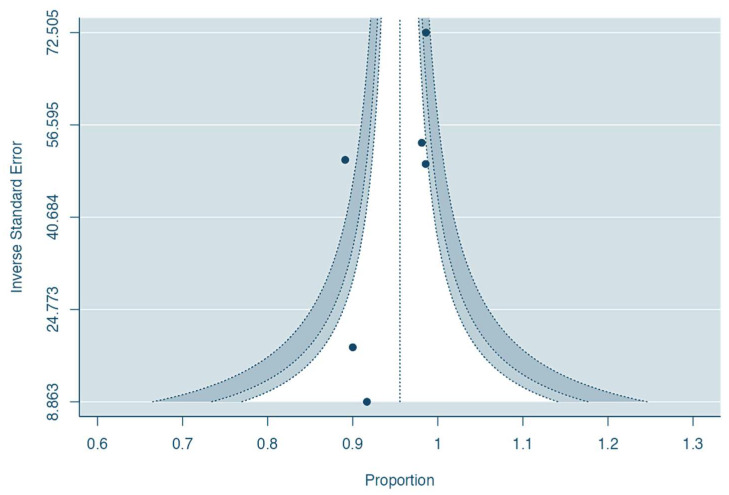
Contour-enhanced funnel plot evaluating the risk of bias for meta-analysis of the pooled rate of satisfaction following breast aesthetic surgery with P4HB (inverse standard error).

The funnel plot graphic for the pooled rate of satisfaction following aesthetic procedures with P4HB suggested no evidence of publication bias, which was further supported with an Egger’s test meta-regression model (*p* = 0.38) (Figure 8). The fail-safe N calculations estimated that 21,775 null-result studies would be required to nullify the observed satisfaction effect.

## 4. Discussion

P4HB exhibits predictable monofilament characteristics, rapid cellular integration (95% within seven days), and prolonged mechanical strength (3–5x after resorption) [35]. It accelerates tissue regeneration and achieves up to three to four times the tensile strength of native tissue [12,34]. Complete resorption occurs within 18–24 months through hydrolysis, minimizing long-term foreign body reactions [34]. Additionally, its capacity to promote collagen deposition and vascularization supports neo-fibrous tissue formation, which may improve surgical outcomes [12,34]. Compared to Vicryl, polydioxanone II, and Mosnocryl, P4HB has demonstrated superior in vivo strength retention [2,17]. Additionally, P4HB exhibits both flexibility and durability, with tensile strength potentially surpassing that of polypropylene sutures [2,17].

Beyond their clinical utility, P4HB-based scaffolds such as GalaFLEX™ represent a cost-effective alternative to ADMs, offering comparable outcomes at a significantly reduced cost [17,31]. Movassaghi reported that while the use of ADM would cost approximately USD 3500 per unit, the equivalent GalaFLEX scaffold was priced at USD 750 per unit, resulting in substantial cost savings without compromising clinical efficacy.

P4HB has shown usefulness in offering mechanical support for both reconstructive and aesthetic breast procedures, facilitating a personalized approach. In breast revision surgery, particularly in cases of implant malposition requiring capsulorrhaphy or neosubpectoral pocket formation, P4HB serves as a structural reinforcement against implant weight. Additionally, its application in primary augmentation may help prevent implant displacement [33,36]. When used for mastopexy, reduction, or augmentation, the scaffold’s ability to enhance the bio-integration between glandular tissue and the breast adipo-cutaneous flap could improve tissue adherence, potentially contributing to long-term stability and reducing the recurrence of ptosis [34]. However, surgeons should avoid excessive mesh fixation, as it has been associated with rare cases of breast constriction, which may occasionally necessitate surgical loosening of the scaffold for correction [30].

In direct-to-implant reconstruction, Karp et al. described the “empanada” technique, where GalaFLEX™ is wrapped around the implant to create a pseudotextured interface that enhances stability during the early postoperative period [28]. The scaffold–implant construct functions similarly to tissue expander tabs, ensuring secure positioning within the breast pocket [28]. Sigalove et al. proposed a combined approach for two-stage breast reconstruction utilizing ADM at the lower pole to facilitate expansion, while employing GalaFLEX at the upper pole to maintain pocket integrity and prevent inferior stretch deformities [12].

The use of P4HB scaffolds in aesthetic breast surgery has demonstrated long-term stability in breast shape and projection. Bistoni et al. reported minimal elongation of the SN-N distance following subfascial augmentation mastopexy (*p* = 0.242), with no significant correlation to changes in breast volume (~10%) or N-IMF elongation (8.04%) [32]. Notably, three years post-surgery, Bistoni et al. observed only a slight N-IMF elongation (9.44%), despite the complete resorption of P4HB at one year, when the N-IMF elongation was 8.6% [32]. Consequently, it can be suggested that lower pole support at one year postoperatively and afterward was primarily dependent on neovascularized connective tissue ingrowth rather than the P4HB scaffold itself [37,38]. However, this hypothesis requires further histologic validation.

Similarly, Buccheri et al. found that patients undergoing mastopexy without mesh reinforcement exhibited a significant increase in N-IMF distance (*p* < 0.05), leading to upper pole volume loss and glandular descent, whereas those with P4HB scaffolds showed improved lower pole support with similar complication rates [1]. In the same line with these findings, Cagli et al. reported less inferior pole elongation in patients undergoing breast reduction/mastopexy with GalaFLEX compared to No-GalaFlex controls (5.6% vs. 12% at 12 months) [34]. Of note, although larger implants are generally believed to increase N-IMF stretch, Bistoni et al. found no significant correlation between implant size and N-IMF elongation in patients who received GalaFLEX reinforcement [32].

Clinical evaluations have shown high satisfaction rates independently from the length of follow-up with P4HB in cosmetic procedures. Sinclair and Adams reported an average satisfaction score of 3.42 out of 4, with no significant variation across different follow-up periods [33]. Scores remained stable over time, with patients at 1–2.5 years (3.4), 2.5–5 years (3.4), and 5–9 years of follow-up (3.5) reporting comparable satisfaction levels [32,33].

In prepectoral breast reconstruction, P4HB differs from ADMs and is generally not utilized for contour enhancement, often leading to visible rippling. Although the absence of pectoral muscle coverage in prepectoral techniques predisposes patients to rippling, fat grafting is commonly employed to mitigate this issue [3,4,17]. Movassaghi et al. reported performing fat grafting in 94% of their cases to address rippling [17]. Conversely, some studies propose that the structural rigidity of GalaFLEX™ may reduce implant wrinkling, potentially minimizing the need for fat grafting [12]. Further research is required to substantiate these findings.

Currently, there is no evidence contraindicating the use of autologous fat grafting following P4HB scaffold placement. In this setting, fat grafting following alloplastic breast reconstruction, including cases involving P4HB scaffold placement, may be indicated for the correction of contour deformities, volume asymmetry, radiation-induced soft tissue damage, or skin atrophy. To ensure adequate tissue healing and implant stabilization, a safe interval for fat grafting is typically between 3 to 6 months after implant placement. In patients who have undergone radiotherapy, a longer interval of at least 6 months post-radiation is recommended to minimize complications and optimize graft retention.

P4HB is a macro-porous monofilament scaffold designed to facilitate vascularized tissue integration, progressively strengthening the construct as it degrades [2,17]. Its monofilament composition and resorbable properties may lower the risk of infection compared to braided, multifilament, or permanent polymer meshes [2,17]. Clinical observations by Movassaghi et al. suggest infection resistance, as P4HB remained adherent in cases requiring tissue expander removal, even within 10 days postoperatively [17]. Notably, mesh removal was unnecessary, and periprosthetic infections resolved following expander explantation without additional interventions [17].

Given its antimicrobial characteristics, some authors, including Sinclair and Adams, proposed managing exposed GalaFLEX™ with local scaffold excision and wound care rather than complete removal [33,39]. In cases of wound dehiscence or delayed healing, Bistoni et al. reported no significant lower pole stretching, possibly due to the structural support provided by the scaffold, even in patients with compromised soft tissue [32]. Our meta-analysis found that the use of P4HB in breast reconstruction was associated with a reduction in wound-related complications. However, potential biases, such as the use of device-based perfusion assessment, negative pressure therapy, or the selection of more suitable surgical candidates, may account for these findings [12].

A review of breast reconstruction cases utilizing GalaFLEX™ reported a 10.3% explantation rate, consistent with findings from prepectoral breast reconstruction involving ADMs [3,4,17]. Higher explantation rates were observed in patients with a history of radiation therapy, advanced age, elevated BMI, smoking, or larger mastectomy specimen sizes, aligning with previous reports [17,40,41].

Regardless of the surgical technique, proper placement of the P4HB scaffold is essential to prevent folding, as any central areas of overlapping material may resist resorption [17,33]. Sinclair and Adams documented a case where an encapsulated fragment remained palpable long-term, necessitating surgical removal [33]. Palpability has been reported with GalaFLEX™, primarily due to excess matrix, which, given its rigidity, cannot conform smoothly and may present as raised lumps [12]. To avoid this issue, excess material should be trimmed during placement [12]. Nonetheless, patients should also be informed that any palpable areas are typically temporary and resolve as the scaffold undergoes resorption [12].

Synthetic matrices, including absorbable polyglactin, and long-term absorbable and non-absorbable titanium-coated polypropylene mesh, have been associated with higher capsular contracture rates compared to biologic alternatives [29,42]. Specifically, reports on capsular contracture incidence with P4HB are inconsistent. Diffley et al. identified a significantly increased risk of capsular contracture with GalaFLEX™ (OR 7.39, *p* = 0.001) compared to FlexHD PP [29]. However, this study had a major limitation in sample size imbalance (GalaFLEX™, n = 21; FlexHD PP, n = 192), raising concerns about the reliability of logistic regression analysis, which typically requires larger sample sizes for validity [29,43]. Conversely, in that same study, GalaFLEX™ was associated with a significantly lower risk of explantation due to capsular contracture (OR 0.0, *p* < 0.001), seroma requiring surgical drainage (OR 0.0, *p* < 0.001), and long-term complications (OR 0.0, *p* < 0.001) when compared to FlexHD [29]. These findings suggest varying degrees of capsular contracture rates and complication severity between the groups [29].

The anti-inflammatory properties of P4HB may contribute to a reduced incidence of capsular contracture [44,45]. Studies indicate that P4HB elicits a stronger and faster M_2_ macrophage response than polypropylene mesh, TIGR, GORE BIO-A, and acellular porcine dermis [46]. Consistent with this, Bistoni et al. found no instances of capsular contracture in a series where artificial intelligence was employed for outcome assessment [32]. The absence of grade III-IV contractures supports the hypothesis that P4HB may offer a protective effect, similar to ADMs and certain synthetic meshes in breast reconstruction [8,47,48]. Movassaghi reported a low overall incidence of capsular contracture (1%; 2/194 breasts), with all cases classified as grade II and no occurrences of grade III or IV contracture [17].

Seroma formation can result from both patient-specific and surgical factors. Karp et al. advocate for the GalaFLEX empanada technique, which secures the implant within the prepectoral pocket, minimizing micromovements and reducing the likelihood of delayed seroma formation [28]. This approach has been reported to enhance implant stability in direct-to-implant prepectoral breast reconstruction [28,49]. Regarding imaging safety, P4HB does not compromise mammography quality [30,33]. Furthermore, GalaFLEX™ is generally not visible on ultrasound and does not interfere with diagnostic imaging [30,33]. Follow-up schedules and clinic visit protocols may vary depending on the specific indication for P4HB use in breast surgery and the surgical technique employed.

Despite the numerous advantages associated with this synthetic scaffold, its contraindications remain consistent with those of other breast meshes and scaffolds. Absolute contraindications include active localized or systemic infection, current breast cancer, allergy to Tetracycline hydrochloride or Kanamycin sulfate, and pregnancy or breastfeeding. Relative contraindications comprise a history of radiation therapy to the breast or chest wall, poorly controlled diabetes mellitus, active tobacco use, and a personal history of breast implant-associated anaplastic large-cell lymphoma (BIA-ALCL) or breast implant-associated squamous cell carcinoma (BIA-SCC).

### 4.1. Limitations

This study has several limitations. Retrospective studies are inherently prone to selection bias, recall bias, and missing data, which can compromise the reliability of the findings. Case series, in particular, often lack control groups, limiting the ability to establish causality or compare outcomes effectively. No randomized controlled trials were found in the literature. Additionally, heterogeneity in study design, patient populations, and outcome measures can make it challenging to draw definitive conclusions or perform meta-analyses. The reliance on previously collected data also restricts the ability to control for confounding variables, potentially affecting the validity and generalizability of the results. The current body of literature on this topic is sparse and generally of low methodological quality. Many of the included studies lacked adequate follow-up data, and long-term outcomes were infrequently reported. Short-term follow-up in multiple studies precludes definitive long-term safety evaluation.

Additionally, there was substantial heterogeneity across studies in terms of patient populations, surgeon learning-curve effects, surgical techniques (e.g., augmentation revisions, mastopexy after augmentation), and outcome measures, which limits the ability to draw definitive conclusions from the meta-analysis. Pooling retrospective and prospective studies with substantially different follow-up periods (e.g., 2.8 to 41.9 months) may compromise the reliability of outcome comparisons. Due to substantial heterogeneity in surgical techniques, a meta-analysis of complications associated with P4HB use in aesthetic procedures could not be performed. Methodologically, poor studies tend to exaggerate the overall estimate of treatment effect and may lead to incorrect inferences. Temporal trends in surgical techniques can affect outcome comparability. The influence of the medical industry on outcome reporting may introduce bias, potentially skewing results in favor of sponsored interventions and limiting the objectivity and generalizability of the findings.

### 4.2. Future Directions

To advance the evidence base and facilitate meaningful comparisons across studies, several critical areas warrant attention in future research. The development and adoption of core outcome sets specific to P4HB scaffold evaluation are essential to ensure consistency and relevance in reported outcomes. Additionally, the implementation of standardized surgical technique protocols will enable more accurate comparative assessments and reduce methodological variability. Establishing minimum follow-up duration requirements is also necessary to support definitive evaluations of long-term safety and performance (>24–36 months). Finally, the standardization of patient-reported outcome measures (PROMs) is crucial to capture the patient perspective consistently and meaningfully across studies. Addressing these needs will enhance the quality, reproducibility, and clinical utility of future research in this area.

## 5. Conclusions

The use of P4HB scaffold in breast reconstruction and aesthetic surgery demonstrates promising outcomes, particularly in reducing wound-related complications. Studies on breast reconstruction indicate optimal satisfactory using patient-reported outcomes. Complication rates for breast reconstruction, including infection and seroma, were relatively low, though some heterogeneity was observed. In aesthetic procedures, P4HB scaffold contributed to improved long-term breast shape stability and high patient satisfaction. Despite these positive findings, further research is needed to confirm long-term benefits and optimize surgical techniques. Overall, P4HB scaffold appears to be a valuable adjunct in personalized breast surgery, enhancing both reconstructive and aesthetic outcomes.

## Figures and Tables

**Table 1:** Demographic and clinical characteristics of studies reporting outcomes of breast reconstruction with P4HB.

Study	Patients	Breasts	Mesh	Age	BMI	Smokers	Diabetes	HTN	Follow up (Mo.)	Neo Ch	Preop Radiation	Adj Ch	Adj Radiation
Sigalove et al., 2022 [12]	135	250	P4HB + AlloDerm	53.7 ± 12	27.5 ± 6.5	1 (0.7%)	26 (19.3%)	30 (22.2%)	15 ± 7.8	23 (17%)	4 (1.6%)	6 (4.4%)	19 (7.6%)
	128	249	AlloDerm Alone	51.2 ± 12.7	29.9 ± 7.4	5 (3.9%)	28 (21.9%)	33 (25.8%)	41.9 ± 12	36 (28.1%)	4 (1.6%)	2 (1.6%)	16 (6.4%)
Chen et al., 2023 [16]	220	161	No Mesh †	49.5 ± 11.4	24.0 ± 5.14	48 (29.8%)	6 (3.7%)	N/A	N/A	N/A	N/A	N/A	14 (8.7%)
		122	ADM	46.8 ± 11.5	22.3 ± 3.30	28 (23.0%)	3 (2.5%)	N/A	N/A	N/A	N/A	N/A	20 (16.4%)
		96	P4HB	50.9 ± 11.9	23.9 ± 6.80	27 (28.1%)	1 (1.0%)	N/A	N/A	N/A	N/A	N/A	11 (11.5%)
		14	No Mesh ∆	42.4 ± 12.4	24.4 ± 7.76	3 (21.4%)	1 (7.1%)	N/A	N/A	N/A	N/A	N/A	1 (7.1%)
Movassaghi et al., 2024 [17]	105	194	P4HB	48.5 (28–77)	27.5 (18.3–44)	4 (3.8%)	8 (7.6%)	21 (20%)	24.6 (9.24–41.04)	23 (21.9%)	6 (3.1%)	19 (18.1%)	22 (11.3%)
Karp et al., 2025 [28]	42	75	P4HB	53 ± 12	24 ± 5	5 (12%)	N/A	N/A	2.83 (0.25–15.6)	N/A	N/A	N/A	N/A
Diffley et al., 2025 [29]	72	122	AlloDerm™	51.4 ± 11.2	28.5 ± 6.5	4 (5.6%)	12 (16.7%)	N/A	18.3 ± 13.7	17 (13.9%)	11 (9.0%)	26 (21.3%)	11 (9.0%)
	114	192	FlexHD^®^	51.1 ± 12.7	27.6 ± 6.9	6 (5.3%)	9 (7.89%)	N/A	9.2 ± 8.1	46 (24.0%)	18 (9.4%)	53 (27.6%)	18 (9.4%)
	15	22	DermACELL	56.5 ± 12.3	26.8 ± 4.9	2 (13.3%)	2 (13.3%)	N/A	15.7 ± 12.2	3 (13.6%)	0 (0.0%)	1 (4.5%)	0 (0.0%)
	12	21	P4HB	44.8 ± 9.1	29.8 ± 4.9	1 (8.3%)	2 (16.7%)	N/A	14.8 ± 9.03	10 (47.6%)	3 (14.3%)	5 (23.8%)	3 (14.3%)
	12	22	Meso BioMatrix	54.3 ± 11.2	28.8 ± 4	1 (8.3%)	0 (0.0%)	N/A	8.3 ± 5.6	1 (4.5%)	4 (18.2%)	5 (22.7%)	4 (18.2%)
	17	25	Autologous Dermal Flap	51.1 ± 12.8	32.2 ± 7.2	0 (0.0%)	1 (5.9%)	N/A	9.9 ± 8.2	8 (32.0%)	5 (20.0%)	10 (40.0%)	5 (20.0%)

ADM, acellular dermal matrix; Adj; adjuvant; BMI, body mass index; Ch, chemotherapy; HTN, hypertension; N/A, not available; Neo, neoadjuvant; Mo., months. † Prepectoral. ∆ Total submuscular.

**Table 2:** Demographic and clinical characteristics of studies reporting outcomes of aesthetic breast surgery with P4HB.

Study	Patients	Mesh	Age	BMI	Smokers	Diabetes	HTN	Follow Up (Mo.)
Adams, Baxter, et al., 2018 [30]	62	P4HB	42.4 ± 9.4	24.7 ± 2.9	0 (0%)	0 (0%)	3 (4.8%)	N/A
Nair & Mills, 2019 [18]	5	P4HB	46.2 ± 12.44	22.4 ± 2.83	N/A	N/A	N/A	15.3 ± 6.6
Buccheri et al., 2023 [8]	34	P4HB	38.2 ± 10.44	23.4 ± 2.83	N/A	N/A	N/A	12 (6–28)
Tomouk & Georgeu, 2023 [31]	6	P4HB	44.6 (32–68)	N/A	N/A	N/A	N/A	36–60
Bistoni et al., 2024 * [32]	72	P4HB	35.6 (18–59)	22.3 (18–33)	8 (11.1%)	N/A	N/A	24.8 (12–45)
Sinclair & Adams, 2024 [33]	248	P4HB	38.2	N/A	N/A	N/A	N/A	34.8 (12–111.6)
Buccheri et al., 2025 * [1]	30	P4HB	32.17 ± 3.24	23.233 ± 2.002	7 (23.3%)	0 (0%)	N/A	12
	30	No Mesh	32.2 ± 3.691	22.647 ± 2.224	5 (16.6%)	1 (3.3%)	N/A	12
Cagli et al., 2024 [34]	5	P4HB	N/A	N/A	N/A	N/A	N/A	N/A
	10	No Mesh	N/A	N/A	N/A	N/A	N/A	N/A

BMI, body mass index; HTN, hypertension; N/A, not available; Mo., months. * Prospective studies.

**Table 3:** Surgical characteristics and outcomes of breast reconstruction with P4HB.

Study	Patients	Breasts	Mesh	Laterality	Technique	Incision Pattern	Plane	Final Implant Size (cc)	Surgical Time (min)
Sigalove et al., 2022 [12]	135	250	P4HB + AlloDerm	Bilateral: 115 (85.2%) Unilateral: 20 (14.8%)	Two-stage §	N/A	Pre-pectoral	N/A	N/A
	128	249	AlloDerm Alone	Bilateral: 121 (94.5%) Unilateral: 7 (5.5%)		N/A	Pre-pectoral	N/A	N/A
Chen et al., 2023 [16]	220	161	No Mesh	N/A	Two-stage	N/A	Prepectoral	N/A	N/A
		122	ADM	N/A		N/A	Dual-Plane ADM	N/A	N/A
		96	P4HB	N/A		N/A	Dual-Plane P4HB	N/A	N/A
		14	No Mesh	N/A		N/A	Total Submuscular	N/A	N/A
Movassaghi et al., 2024 [17]	105	194	P4HB	Bilateral 89 (84.7%) Unilateral 16 (15.2%)	Two-stage §	Inferolateral for non-ptotic Mastopexy for ptotic	Pre-pectoral	573 ± 153.4	75 † 105 ∆
Karp et al., 2025 [28]	42	75	P4HB	N/A	Direct-to-implant §	N/A	Pre-pectoral	N/A	N/A
Diffley et al. 2025 [29]	72	122	AlloDerm™	Bilateral: 50 (69.4%) Unilateral: 22 (30.6%)	Direct-to-implant §	N/A	Prepectoral: 80 (65.6%) Subpectoral: 42 (34.4%)	N/A	N/A
	114	192	FlexHD^®^	Bilateral: 78 (68.4%) Unilateral: 36 (31.6%)		N/A	Prepectoral: 180 (93.8%) Subpectoral: 12 (6.3%)	N/A	N/A
	15	22	DermACELL	Bilateral: 6 (40.0%) Unilateral: 9 (60.0%)		N/A	Prepectoral: 9 (40.9%) Subpectoral: 13 (59.1%)	N/A	N/A
	12	21	P4HB	Bilateral: 9 (75.0%) Unilateral: 3 (25.0%)		N/A	Prepectoral: 20 (95.2%) Subpectoral: 1 (4.8%)	N/A	N/A
	12	22	Meso BioMatrix^®^	Bilateral: 10 (83.3%) Unilateral: 2 (16.7%)		N/A	Prepectoral: 20 (90.9%) Subpectoral: 2 (9.1%)	N/A	N/A
	17	25	Autologous Dermal Flap	Bilateral: 6 (35.3%) Unilateral: 11 (64.7%)		N/A	Prepectoral: 15 (60.0%) Subpectoral: 10 (40.0%)	N/A	N/A

Min, minutes; N/A, not available. § Explicitly clarified in the study that authors employed immediate breast reconstruction. † Time for procedures when using inferolateral incision. ∆ Time for procedures when using mastopexy incision. In Sigalove et al., 2022, patients who received P4HB-AlloDerm had significant lower BMI (*p* = 0.006), lower rates of preoperative chemotherapy (*p* = 0.031), lower rates of skin-reducing mastectomy (*p* < 0.001), and lower rates of bilateral reconstructions (*p* = 0.013) [12]. In Diffley et al., 2025, the percentage of bilateral versus unilateral reconstruction (*p* = 0.014), the implant location (prepectoral versus subpectoral *p* < 0.001), and the rate of neoadjuvant chemotherapy (*p* < 0.001) were significantly different among groups [29].

**Table 4:** Surgical characteristics and outcomes of aesthetic breast surgery with P4HB.

Study	Patients	Mesh	Indication	Specimen Weight (gr)	Technique	Implant Plane	Final implant Size (cc)	Surgical Time (min)	Pedicle
Adams, Baxter, et al., 2018 [30]	62	P4HB	Breast ptosis	R/: 122.1 ± 110.4 (n = 31) L/: 131.5 ± 107.8 (n = 34)	Breast Reduction Mastopexy	N/A	N/A	N/A	Superior: 8 (12.9%) Inferior: 39 (62.9%) Central: 21 (33.9%) Other: 2 (3.2%)
Nair & Mills, 2019 [18]	5	P4HB	Capsular contracture: 5 (100%) Implant ripping: 1 (20%)	N/A	Capsulectomy: 3 (60%) Capsulotomy: 1 (20%) Capsulorrhaphy: 1 (20%)	Subpectoral: 4 (80%) Subglandular: 1 (20%)	540 ± 157	N/A	N/A
Buccheri et al., 2023 [8]	34	P4HB	Breast Augmentation Revision	N/A	Capsulectomy with mastopexy: 12 (35.3%) Capsulectomy w/o mastopexy: 22 (64.7%)	Subglandular	345 (325–440)	160 (140–180)	N/A
Tomouk and Georgeu, 2023 [31]	6	P4HB	Breast Augmentation Revision	N/A	Capsulectomy with mastopexy: 4 (66.6%) Capsulectomy w/o mastopexy: 2 (33.33%)	Subglandular	244 (170–320)	N/A	N/A
Bistoni et al., 2024 [32]	72	P4HB	Breast Augmentation	286.4 (70–653)	Augmentation Mastopexy - Primary: 46 - Secondary: 26	Subfascial	294 (175–510)	N/A	N/A
Sinclair & Adams, 2024 [33]	248	P4HB	Breast ptosis: 167 (68.2%) Capsular contracture: 30 (12.2%) Implant malposition: 29 (11.8%) Hypomastia: 10 (4.1%) Macromastia: 12 (4.9%)	N/A	Mastopexy: 88 (35.5%) Augmentation-mastopexy: 82 (33.1%) Implant exch. with site change: 30 (12.1%) Neosubpectoral pocket creation: 17 (6.9%) Capsulorrhaphy: 12 (4.8%) Breast reduction: 10 (4.0%) Implant exch. with capsulectomy: 7 (2.8%) Primary breast augmentation: 2 (0.8%)	N/A	N/A	N/A	N/A
Buccheri et al., 2025 [1]	30	P4HB	Regnault grade III breast ptosis	N/A	Mastopexy §	N/A	N/A	121.5 ± 9.2	Inferior pedicle (Dermo-adipose)
	30	No Mesh		N/A	Mastopexy §	N/A	N/A	122.5 ± 10.2	Inferior pedicle (Dermo-adipose)
Cagli et al., 2024 [34]	5	P4HB	Grade III ptosis	N/A	Breast Reduction §	N/A	N/A	N/A	Inferior pedicle (Dermo-adipose)
	10	No Mesh		N/A	Breast Reduction §	N/A	N/A	N/A	Inferior pedicle (Dermo-adipose)

cc, cubic centimeter; exch., exchange; min, minute; L/, left; gr, grams; P4HB, poly-4-hydroxybutyrate; R/, right; w/o, without. § Explicitly clarified in the study that authors used Wise-pattern incision.

## Data Availability

Data for this manuscript is publicly available.

## Supplementary Materials

The following supporting information can be downloaded at: https://www.mdpi.com/article/10.3390/jpm15080368/s1, Supplemental Digital Content S1: Postoperative complications following breast reconstruction with P4HB; Supplemental Digital Content S2: Postoperative complications following aesthetic breast surgery with P4HB.

## Abbreviations

The following abbreviations are used in this manuscript:

P4HB	poly-4-hydroxybutyrate
ADMs	acellular dermal matrices
IMF	inframammary fold
OR	odds ratio
IQR	interquartile range
BMI	Body Mass Index
RTOR	return to the operating room
MQAT	Methodological Quality Assessment Tool
Q3	upper quartile
ROBINS-I	Risk Of Bias In Non-randomized Studies-of Interventions
OCEBM	Oxford Centre for Evidence-Based Medicine
N-IMF	nipple-to- inframammary fold distance
SN-N	sternal notch to the nipple
