# Supplementary material for: Poly-4-Hydroxybutyrate as a Novel Biomaterial in Personalized Breast Surgery: A Systematic Review and Meta-Analysis
Source: J Pers Med. 2025 Aug 12;15(8):368. doi: 10.3390/jpm15080368 (PMC12387284; doi:10.3390/jpm15080368)
Supplement: Supplementary file 1 [file jpm-15-00368-s001.zip › Supplemental Digital Content.pdf]

Supplemental Digital Content S1: Postoperative complications following breast reconstruction with P4HB.

Study	Breasts	Mesh	Infection	Seroma	Skin Flap Necrosis	Delayed wound Healing	Implant exposure	Explantation	RTOR	NAC Excision	Hematoma	Revision	LDF	Contracture	Implant Malposition
Sigalove et al., 2022	250	P4HB + AlloDerm	5 (2.0%)	8 (3.2%)	3 (1.2%)	N/A	4 (1.6%)	8 (3.2%)	13 (5.2%)	N/A	N/A	N/A	N/A	2 (0.8%)	N/A
	249	AlloDerm Alone	4 (1.6%)	7 (2.8%)	13 (5.2%)	N/A	3 (1.2%)	4 (1.6%)	9 (3.6%)	N/A	N/A	N/A	N/A	2 (0.8%)	N/A
Chen et al., 2023	161	No Mesh †	21 (13.0%)	N/A	19 (11.8%)	N/A	N/A	N/A	N/A	N/A	N/A	75 (46.6%)	N/A	49 (30.4%)	N/A
	122	ADM	16 (13.1%)	N/A	16 (13.1%)	N/A	N/A	N/A	N/A	N/A	N/A	57 (46.7%)	N/A	42 (34.4%)	N/A
	96	P4HB	7 (7.3%)	N/A	4 (4.2%)	N/A	N/A	N/A	N/A	N/A	N/A	40 (41.7%)	N/A	46 (47.9%)	N/A
	14	No Mesh Δ	2 (14.3%)	N/A	2 (14.3%)	N/A	N/A	N/A	N/A	N/A	N/A	8 (57.1%)	N/A	3 (21.4%)	N/A
Movassaghi et al., 2024	194	P4HB	13 (6.7%)	0 (0.0%)	8 (4.1%)	N/A	7 (3.6%)	20 (10.3%)	20 (10.3%)	7 (3.6%)	0 (0.0%)	5 (4.7%)	3 (1.5%)	2 (1.0%)	2 (1.0%)
Karp et al., 2025	75	P4HB	1 (1.3%)	1 (1.3%)	1 (1.3%)	N/A	1 (1.3%)	2 (2.6%)	2 (2.6%)	N/A	N/A	N/A	N/A	N/A	N/A
Diffley et al. 2025	122	AlloDerm™	20 (16.4%)	7 (5.7%)	11 (9.0%)	N/A	5 (4.1%)	25 (20.5%)	18 (14.8%)	N/A	3 (2.5%)	47 (38.5%)	N/A	11 (9.0%)	2 (1.6%)
	192	FlexHD®	20 (10.4%)	4 (3.2%)	17 (8.9%)	N/A	8 (4.2%)	25 (13.0%)	23 (12.0%)	N/A	8 (4.1%)	60 (31.3%)	N/A	3 (1.6%)	6 (3.1%)
	22	DermACELL	2 (9.1%)	0 (0.0%)	2 (9.1%)	N/A	0 (0.0%)	1 (4.5%)	1 (4.5%)	N/A	0 (0.0%)	7 (31.8%)	N/A	0 (0.0%)	0 (0.0%)
	21	P4HB	1 (4.8%)	0 (0.0%)	0 (0.0%)	N/A	3 (14.3%)	4 (19.0%)	2 (9.5%)	N/A	0 (0.0%)	9 (42.9%)	N/A	0 (0.0%)	0 (0.0%)
	22	Meso BioMatrix®	3 (13.6%)	0 (0.0%)	3 (13.6%)	N/A	4 (18.2%)	4 (18.2%)	2 (9.1%)	N/A	0 (0.0%)	8 (36.4%)	N/A	0 (0.0%)	0 (0.0%)
	25	Autologous Dermal Flap	4 (16.0%)	5 (22%)	2 (8.0%)	N/A	1 (4.0%)	5 (20%)	4 (16.0%)	N/A	1 (4.0%)	7 (28.0%)	N/A	2 (8.0%)	0 (0.0%)

LDF, latissimus dorsi flap; N/A, not applicable; NAC, nipple-areola compex; RTOR, return to the operating room.
† Prepectoral
Δ Total Submuscular

Supplemental Digital Content S2: Postoperative complications following aesthetic breast surgery with P4HB.

Study	Patients	Breasts	Mesh	Infection	Seroma	Skin Flap Necrosis	Hypertrophic Scar	Dehiscence	Delayed wound Healing	Implant exposure	Explantation	RTOR	Palpable Mesh	NAC Excision	Hematoma	Revision	LDF	Contracture	Implant Malposition
Adams, Baxter, et al. 2018	62	N/A	P4HB	4 (6.4%)	0 (0%)	0 (0%)	0 (0%)	7 (11.3%)		0 (0%)	N/A	4 (6.4%)	N/A	0 (0%)	3 (4.8%)	0 (0%)	0 (0%)	N/A	N/A
Nair & Mills 2019	5	10	P4HB	0 (0%)	0 (0%)	0 (0%)	0 (0%)	0 (0%)	0 (0%)	0 (0%)	0 (0%)	0 (0%)	N/A	N/A	0 (0%)	N/A	0 (0%)	0 (0%)	0 (0%)
Buccheri et al. 2023	34	N/A	P4HB	0 (0%)	0 (0%)	0 (0%)	0 (0%)	0 (0%)	0 (0%)	0 (0%)	0 (0%)	0 (0%)	0 (0%)	0 (0%)	0 (0%)	0 (0%)	0 (0%)	0 (0%)	0 (0%)
Tomouk & Georgeu 2023	6	N/A	P4HB	0 (0%)	0 (0%)	0 (0%)	0 (0%)	0 (0%)	0 (0%)	0 (0%)	0 (0%)	0 (0%)	0 (0%)	0 (0%)	0 (0%)	0 (0%)	0 (0%)	0 (0%)	0 (0%)
Bistoni et al., 2024	72	N/A	P4HB	0 (0%)	0 (0%)	0 (0%)	0 (0%)	3 (4.2%)	8 (11.1%)	0 (0%)	0 (0%)	0 (0%)	0 (0%)	0 (0%)	0 (0%)	0 (0%)	0 (0%)	0 (0%)	0 (0%)
Sinclair & Adams, 2024	248	N/A	P4HB	0 (0%)	1 (0.4%)	0 (0%)	5 (2%)	4 (1.6%)	7 (2.8%)	N/A	N/A	7 (2.8%)	1 (0.4%)	N/A	2 (0.8%)	7 (2.8%)	0 (0.0%	1 (0.4%)	3 (1.2%)
Buccheri et al. 2025	30	60	P4HB	0 (0%)	0 (0%)	0 (0%)	0 (0%)	1 (3.33%)	0 (0%)	N/A	N/A	0 (0%)	0 (0%)	0 (0%)	0 (0%)	0 (0%)	0 (0.0%	N/A	N/A
	30	60	No Mesh	0 (0%)	0 (0%)	0 (0%)	0 (0%)	2 (6.66%)	0 (0%)	N/A	N/A	0 (0%)	0 (0%)	0 (0%)	0 (0%)	0 (0%)	0 (0.0%	N/A	N/A
Cagli et al. 2024	5	N/A	P4HB	0 (0%)	0 (0%)	0 (0%)	0 (0%)	0 (0%)	0 (0%)	N/A	N/A	0 (0%)	0 (0%)	0 (0%)	0 (0%)	0 (0%)	0 (0%)	N/A	N/A
	10	N/A	No Mesh	0 (0%)	0 (0%)	0 (0%)	0 (0%)	0 (0%)	0 (0%)	N/A	N/A	0 (0%)	0 (0%)	0 (0%)	0 (0%)	0 (0%)	0 (0%)	N/A	N/A

LDF, latissimus dorsi flap; N/A, not applicable; NAC, nipple-areola compex; RTOR, return to the operating room.
